# Double bucket handle tear of the superior labrum in a young patient presented with shoulder instability treated with arthroscopic debridement: A case report

**DOI:** 10.1016/j.ijscr.2021.105939

**Published:** 2021-04-30

**Authors:** Sultan Khaled Alharbi, Adel Alahaidib, Mouad Alsowaigh, Jawaher Alharbi, Abdulaziz Alahaideb

**Affiliations:** a King Khaled University Hospital, Riyadh, Saudi Arabia; b Prince Mohammed bin Abdulaziz Hospital, Riyadh, Saudi Arabia

**Keywords:** Glenohumeral joint, Shoulder instability, Superior labrum anterior to posterior tears, Double bucket-handle tear, Case report, Shoulder arthroscopy

## Abstract

**Introduction and importance:**

The two major etiologies of shoulder superior labral tears anterior to posterior (SLAP) are traumatic and degenerative processes. Bucket handle tears of the superior labrum represent one-third of labral lesions. However, in this article, we present a double bucket handle tear which has been reported once in the literature.

**Presentation of case:**

A 25-year-old male presented with complaint of chronic pain in his right shoulder with a remote history of traumatic dislocation. Physical examination revealed a positive apprehension test. Shoulder magnetic resonance imaging (MRI) showed a superior labral tear with a Hill-Sach lesion. Arthroscopy showed a double bucket handle tear of superior labrum and mild biceps tendonitis along with Bankart lesion. The tear was resected and the Bankart lesion was repaired followed by supervised physical therapy. Good clinical outcomes in form of resolution of pain and shoulder instability at six months were obtained.

**Discussion:**

SLAP tears are common shoulder lesion that is reported differently in the literature. Arthroscopic studies had reported the incidence between 3.9%-11.8. The diagnosis of such lesion relies on the clinical presentation and imaging. Knesek et al. classified SLAP lesions based on the integrity of the biceps anchor and the type of labral tear (Knesek et al., 2013). The standard treatment of symptomatic SLAP lesions is Arthroscopic debridement. However, non-operative management was described in the literature.

**Conclusion:**

Double bucket handle injuries of the superior labrum are reported in literature once. These lesions can be treated with arthroscopic debridement and Bankart repair and followed by supervised physical therapy.

## Highlights

• Superior labrum Anterior to posterior (SLAP) lesions are common cause of shoulder pain.
• Various causes for SLAP lesions have been described including micro traumatic and non-traumatic (e.g., degenerative).
• The incidence of SLAP tears varies among multiple arthroscopic studies. Snyder et al. reported an incidence of 3.9% to 11.8.
• Most of patients with SLAP lesions present with concurrent shoulder injuries such as partial or complete rotator cuff tears.
• MR arthrography is the best imaging Modality to evaluate the SLAP lesions.
• Arthroscopic SLAP repair remains the gold standard treatment. However, management depends on patient age, functional status.

## 1 Introduction

Since the mid-1980s, Andrews was the first author, who described superior labral pathologies that were recognized as a cause of shoulder pain [1]. After that, Snyder et al. established the current understanding of the pathologic anatomy of Superior labrum Anterior to posterior (SLAP) lesions and developed the initial 4-subtype classification of these lesions [2]. Subsequently, Maffet et al. subdivided the SLAP classification schemes to ultimately delineated ten different types of SLAP tear [3]. The specific etiology of different types of SLAP lesions remains controversial. The two major defined etiologies include (1) Acute Traumatic SLAP Lesions and (2) Attritional SLAP Injuries. The treatment plan for SLAP lesions does not depend solely on the specific type, the patient's age, functional level, and it also plays a major role. In this article, we present a case report of double bucket handle tear of superior labrum for a young patient treated as a case of shoulder instability, which has been reported once in the literature [4]. This case was reported in line with the 2020 SCARE criteria [5].

## 2 Case presentation

**Fig. 1:**
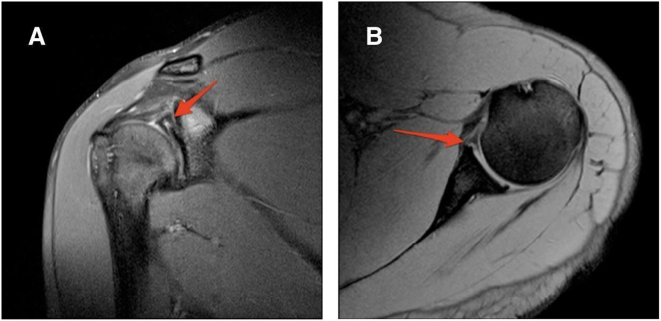
A: Coronal cut of right shoulder MRI showing Bucket-Handle tear of the labrum, Hill-Sach lesion. B: Axial cut of right shoulder MRI showing Bankart lesion.

**Fig. 2:**
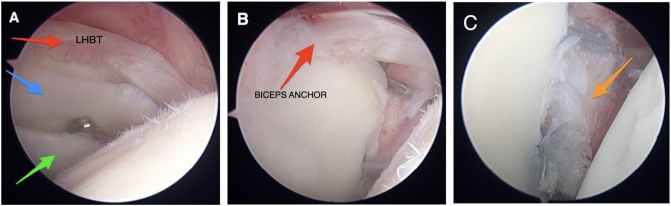
A: Arthroscopic image showing double bucket handle tear (blue and green arrows) and inflamed biceps tendon (red arrow). B: Arthroscopic image showing intact biceps anchor (red arrow). C: Arthroscopic image showing Bankert lesion after repair. (For interpretation of the references to color in this figure legend, the reader is referred to the web version of this article.)

A 25-year-old male student, right hand dominant, was referred to our clinic by a family physician with a major complaint of pain in his right shoulder and feeling of instability. Shoulder pain exacerbated with overhead activities which interfered with his ability to perform weightlifting exercises. The patient sustained shoulder dislocation after falling on an outstretched arm three years ago which was treated conservatively with a closed reduction followed by 2 weeks of immobilization and physical therapy. The patient denied the use of any medications except occasional use of nonsteroidal anti-inflammatory drugs for pain and reported no history of allergies. Family history was unremarkable. Upon physical examination, the patient looked overweight with a body mass index of 28.3. Both shoulder girdles looked symmetrical with no atrophic changes being noticed. Passive range of motion revealed no limitation (anterior elevation, 180°; external rotation, 80°; internal rotation, T10; and abduction, 180°). The pain was elicited by external rotation, generalized ligamentous laxity was not observed. The shoulder apprehension test was positive. Load and shift test Showed an anterior-posterior subluxation of the shoulder. Magnetic resonance imaging was performed, which revealed a right bucket-handle tear of the labrum with intact biceps anchor, a type III SLAP lesion, and a Hill-Sachs lesion (Fig. 1A, B). Based on these findings, surgical intervention was considered necessary for shoulder instability. An elective standard shoulder arthroscopy was performed by a senior shoulder surgeon under general anesthesia in the beach chair position. The intraoperative arthroscopic findings included (1) inflamed biceps tendon with mild fraying (2) Banker lesion (3) a tear of the superior labrum into an upper band and a lower band (double bucket handle tear, however, the biceps anchor was intact (Fig. 2A). The upper and lower bands of the labral tear were debrided (Fig. 2B), and after the preparation of the glenoid rim, two suture anchors were used for labrum fixation. The perioperative course was unremarkable, and the patient was discharged home on the same day. Postoperatively, the patient was placed on an abduction orthosis for six weeks and a wound check was performed at 2 weeks. At 6 weeks follow-up, pendulum, and periscapular muscle activation exercises were performed for restoration of range of motion. Abduction, internal rotation, external rotation, and resistive exercises were not allowed at 10 postoperative weeks. During the early postoperative period, the patient was adherent to the physical therapy protocol and reported no issues. At 6 months follow-up, the patient reported a resolution of pain and absent sense of instability. The patient achieved full range of motion and stable painless shoulder at his final follow-up 2 years after surgery.

## 3 Discussion

The long head of the biceps tendon and superior labrum help to stabilize the humeral head. After the development of shoulder arthroscopic interventions, superior labrum anterior to posterior (SLAP) tears became a well-recognized problem in recent times. It represents a significant cause of shoulder pain especially among athletes involved in repetitive overhead activities.

There was a significant difference in the proportion of SLAP tears found in multiple shoulder arthroscopic studies. Snyder et al. analyzed 700 shoulder arthroscopies and found a SLAP incidence of 3.9% [2]. Maffet et al. identified 84 SLAP-type lesions in 712 shoulders arthroscopies, constituting an incidence rate of 11.8% [3]. Handel-berg et al. reported that 32 (6%) of 530 patients had such a lesion [6]. One review of the clinical presentation of SLAP tears reported that of 544 shoulder arthroscopy procedures performed, 139 (26%) revealed a SLAP lesion [7]. This initial rise in the incidence rate of SLAP repairs performed reached its peak before subsequently declining over the last decade. A study in 2015 reported the adjusted annual incidence rate for SLAP lesions increased from 0.31 cases per 1000 person-years in 2002 to 1.88 cases per 1000 person-years in 2009, with an average annual increase of just over 20% during the study period [8]. Zhang et al. found that there were 25,574 arthroscopic SLAP repairs between 2004 and 2009 [9]. Weber et al. reported an increase in the SLAP repair rate to greater than 10% of shoulder cases reported by the year 2008 [10]. Erickson et al. in 2016 reported that an institutional trend from 2004 to 2014 revealed decreasing rates of total SLAP repairs performed [11].

The pain was the most common clinical complaint reported from 140 patients with superior glenoid labrum injury, with 49% of all patients noting mechanical catching or grinding in their shoulder [12].

The majority of patients present with concurrent shoulder injuries. In a retrospective review of 140 arthroscopically proven SLAP lesions, the reported incidence of associated intra-articular disease included 29% with partial rotator cuff tears, 11% with full rotator cuff tears, 22% with Bankart lesions, and 10% with glenohumeral chondromalacia [12].

SLAP tears are known to be caused by both macro-trauma and micro-trauma, the exact forces which create these lesions remain unknown and therefore also controversial. Multiple mechanisms were described in the literature which includes (1) Compression-type injuries which include a fall onto an outstretched arm with the arm in varying degrees of shoulder abduction, (2) Traction-type injuries which can occur secondary to sudden jerking movements or after lifting heavy objects or after an unexpected pull on the arm and (3) Peel-back mechanism, the twisting that occurs at the base of the biceps transmits torsional forces to the posterosuperior labrum and usually happens in a position of abduction and maximal external rotation [13]. Compression force to the shoulder, usually as the result of a fall onto an outstretched arm, with the shoulder positioned in the abduction and slight forward flexion at the time of the impact, is the most common mechanism of injury that was described by Snyder et al.^2^.

Knesek et al. classified SLAP tears into 4 distinct types. Type 1: Degenerative fraying of the superior labrum, biceps anchor is intact; Type 2: Superior labrum and biceps tendon detachment from glenoid rim; Type 3: Bucket-handle tear of the labrum with intact biceps anchor; Type 4: Bucket-handle tear of labrum extended into the biceps tendon [14]. According to Maffet et al., only 62% of their shoulder series was fitting to Snyder's classification schema and later this classification required some modification [3]. As a result, they described 6 new subtypes.

Diagnosis of the SLAP tears is based on the clinical history, a detailed physical examination, and MRI. MR arthrography is the best imaging technique for evaluating SLAP lesions.

The majority of literature establishes the superiority of MR arthrography over conventional MRI for evaluation of the labrum, reporting sensitivities ranging from 82 to 100%, specificities between 71 and 98%, and accuracies between 83 and 94% [15].

Arthroscopic SLAP repairs remain the gold standard treatment. Multiple studies were conducted to assess the effectiveness of non-operative treatment of superior labral tears. Edwards et al. showed that successful non-operative treatment of superior labral tears results in pain relief and functional improvement compared with pretreatment assessments [16].

The described case in this report presented with recurrent shoulder pain, recurrent anterior shoulder instability, and diagnosed with bucket handle tear of the superior labrum (type III SLAP).

The mechanism of injury was a fall on an outstretched arm. If recurring shoulder pain presents in a patient with shoulder instability in association with a previous history of trauma, this should raise the suspicion of a concomitant SLAP lesion. Many Surgeons missed the diagnosis of SLAP lesion due to not ordering magnetic resonance arthrography. MR arthrography is the best imaging technique to evaluate the SLAP lesions but because of the high incidence of false-positive cases, a detailed correlation with clinical history, and physical examination are the key to diagnosis. Although the current literature does not support that MRI can accurately differentiate all SLAP types. Furthermore, further studies are necessary to compare MR arthrography and shoulder arthroscopy for the classification of SLAP lesions. The ideal treatment for SLAP tears has not been elucidated, several authors have proposed surgical treatment algorithms depending on the specific type of SLAP lesion [17]. The literature is lacking concerning the relative efficacy of the non-operative management of the different types of SLAP lesions. Surgical treatment which includes resection of the unstable bucket-handle tear was considered as the first-line treatment in this case presentation, as conservative treatment is generally unsuccessful in type III SLAP lesion. Utmost care should be taken in determining whether to perform debridement or suture repair for a double bucket handle tear of the superior labrum. Lastly, we suggest adding a double bucket handle as a subcategory in the modified Snyder classification.

In this report, we presented a rare case of arthroscopically confirmed double bucket handle tears of the superior labrum in a patient with anterior shoulder instability and shoulder pain along with a review of the literature.

## 4 Conclusion

SLAP lesions are a common cause of shoulder pain. The double bucket handle tear is a rare type that has been reported once in the literature. In this article, a young patient with a double bucket handle tear of superior shoulder labrum was treated successfully with Arthroscopic debridement and Bankert repair followed by a supervised rehabilitation program. Given the configuration of a double bucket handle tear, we suggest adding it as a subcategory in the currently used classification system such as Snyder classification [2].

## Provenance and peer review

Not commissioned, externally peer-reviewed.

## Consent

Written informed consent was obtained from the patient for publication of this case report and accompanying images. A copy of the written consent is available for review by the Editor-in-Chief of this journal on request.

## Ethical approval

The Institutional Review Board at King Saud University Medical City had been requested to exempt the study from ethical approval.

## Funding

This article received no funding.

## Author contribution

1. Sultan Khaled Alharbi: Data Collection, literature review, and manuscript writing.

2. Adel Alahaidib: Data Collection and manuscript review.

3. Mouad Alswaiegh: References review.

4. Jawaher Alharbi: Data Collection, literature review and manuscript writing.

5. Abdualaziz Alahaideb: Manuscripts writing and review.

## Guarantor

Abdulaziz Alahaideb, MBBS, FRCSC.

Professor of Orthopedic Surgery, King Khaled University Hospital.

King Khalid Road, King Saud University, Riyadh, Saudi Arabia.

## Research registration

Not applicable as there was no new surgical technique or new equipment.

## Declaration of competing interest

No conflict of interest in relevance to this article was presented.
